# High efficiency error suppression for accurate detection of low-frequency variants

**DOI:** 10.1093/nar/gkz474

**Published:** 2019-05-25

**Authors:** Ting Ting Wang, Sagi Abelson, Jinfeng Zou, Tiantian Li, Zhen Zhao, John E Dick, Liran I Shlush, Trevor J Pugh, Scott V Bratman

**Affiliations:** 1 Department of Medical Biophysics, University of Toronto, Toronto, Ontario, Canada; 2 Princess Margaret Cancer Centre, University Health Network, Toronto, Ontario, Canada; 3 Ontario Institute for Cancer Research, Toronto, Ontario, Canada; 4 Department of Molecular Genetics, University of Toronto, Toronto, Ontario, Canada; 5 Department of Immunology, Weizmann Institute of Science, Rehovot, Israel; 6 Department of Radiation Oncology, University of Toronto, Toronto, Ontario, Canada

## Abstract

Detection of cancer-associated somatic mutations has broad applications for oncology and precision medicine. However, this becomes challenging when cancer-derived DNA is in low abundance, such as in impure tissue specimens or in circulating cell-free DNA. Next-generation sequencing (NGS) is particularly prone to technical artefacts that can limit the accuracy for calling low-allele-frequency mutations. State-of-the-art methods to improve detection of low-frequency mutations often employ unique molecular identifiers (UMIs) for error suppression; however, these methods are highly inefficient as they depend on redundant sequencing to assemble consensus sequences. Here, we present a novel strategy to enhance the efficiency of UMI-based error suppression by retaining single reads (singletons) that can participate in consensus assembly. This ‘Singleton Correction’ methodology outperformed other UMI-based strategies in efficiency, leading to greater sensitivity with high specificity in a cell line dilution series. Significant benefits were seen with Singleton Correction at sequencing depths ≤16 000×. We validated the utility and generalizability of this approach in a cohort of >300 individuals whose peripheral blood DNA was subjected to hybrid capture sequencing at ∼5000× depth. Singleton Correction can be incorporated into existing UMI-based error suppression workflows to boost mutation detection accuracy, thus improving the cost-effectiveness and clinical impact of NGS.

## INTRODUCTION

High-throughput sequencing technologies have revolutionized genetic and biomedical research by uncovering alterations responsible for the development of disease. Although considerable progress has been made toward germline and somatic variant detection, identification of variants at lower allele frequencies remains hindered by sequencing errors and technical artefacts. This has numerous implications in oncology, particularly in liquid biopsy applications, where tumour DNA fragments may be present at frequencies <0.01% (1,2). Sensitive detection is difficult in these scenarios as sequencer error rates average ∼0.1–1% (3,4).

**Figure 1.**
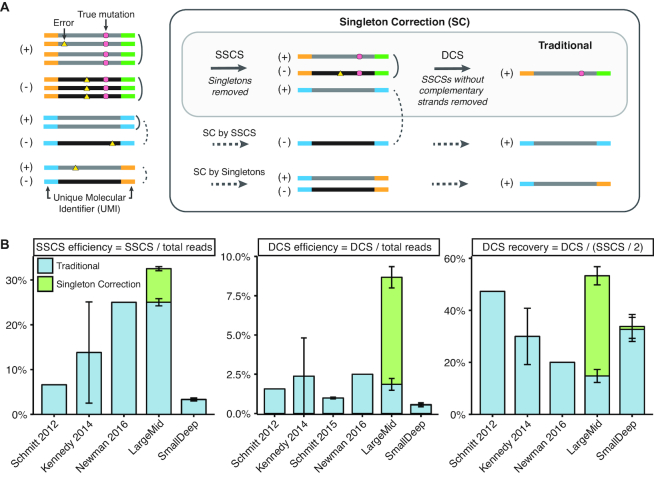
Singleton Correction improves traditional duplex UMI methods. (**A**) Singleton correction (SC) can be achieved through two strategies. (i) In the absence of redundant reads, singletons derived from complementary strands can be used to correct one another for *SC by Singletons*. (ii) If PCR duplicate reads are present only for one strand, they are first collapsed to form a single strand consensus sequence (SSCS). This can be subsequently used to correct the singleton of the complementary strand for *SC by SSCS*. Uncollapsed reads are not an accurate representation of molecular diversity and contain polymerase, sequencer, and oxidation errors. Traditional UMI methods of error suppression are restricted to molecules with redundant reads. Singleton Correction expands error suppression to duplex-matched singletons and enables error correction for a greater number of reads. (**B**) Comparisons of traditional duplex UMI methods from the indicated publications (8–10) and from this study (LargeMid, *n* = 12 libraries; SmallDeep, *n* = 8 libraries). Plot shows SSCS and duplex consensus sequence (DCS) efficiency and recovery for methods with traditional duplex UMI processing or with Singleton Correction. Efficiency is an assessment of over-sequencing relative to unique molecules, whereas recovery is an estimate of molecular retention after sequencing. Data are presented as mean ± S.D.

A promising strategy to suppress errors uses unique molecular identifiers (UMIs) to compare multiple reads derived from the same DNA fragment (Figure 1A) (5–7). Errors that are found in individual reads are removed, and only variants present across all redundant reads are retained to form a single-strand consensus sequence (SSCS). In addition, strand-aware duplex correction is needed to eliminate artefacts from oxidative damage; duplex consensus sequences (DCSs) retain only true variants found on both strands of a fragment by comparing complementary SSCSs (Figure 1A) (8–10). While duplex methods allow for greater error suppression (Supplementary Figure S1), the efficiency of DCS recovery from SSCSs is poor (15–47%, Figure 1B) and reliant on sequencing coverage (Supplementary Figure S2).

A major limitation of current UMI-based error correction methods is the dependence on redundant sequencing (11). This results in poor efficiency with low yield of unique sequences despite high sequencing costs. These inefficiencies are further magnified in duplex UMI methods, where both strands of a molecule must be redundantly sequenced (8–10). This is problematic, as uneven sequencing often arises from amplification biases, stochastic sampling, and inadequate coverage (11–13). These factors limit the applicability of duplex correction to only 0.5–2.5% of sequenced reads (Figure 1B). Furthermore, current UMI-based strategies do not utilize error suppression for single reads (singletons) that have not been redundantly sequenced. This is detrimental as singletons may account for over half of all reads in a moderately deep sequenced sample (defined as ∼1000×–10 000× coverage in this study).

To address these limitations, we developed a ‘Singleton Correction’ methodology that enables error suppression in singletons (Figure 1A). By utilizing the large number of singletons present in hybrid capture deep sequencing data, Singleton Correction allows dramatically more sequences to be corrected. Unlike traditional UMI methods that are restricted to redundant reads, our method also eliminates errors in singletons using reads from the complementary strand. Here, we analyzed a combination of cell line and clinical samples and found that Singleton Correction consistently improved the efficiency of traditional duplex correction methods and increased sensitivity while maintaining high specificity for calling low-allele-frequency variants.

## MATERIALS AND METHODS

### Targeted panel design

We constructed hybrid capture panels targeting genomic footprints representing two different experimental strategies. A 13 kb panel we named ‘SmallDeep’ was intended for ultra-deep sequence coverage and encompassed exons of five genes (*KRAS, NRAS, BRAF, EGFR* and *PIK3CA*) important in the mitogen-activated protein kinase (MAPK) pathway. We have previously used this panel for cell-free DNA sequencing analysis in multiple myeloma (14). A 1.2 Mb panel we named ‘LargeMid’ was intended for moderately deep sequence coverage and encompassed exons from 260 leukemia associated genes (xGen^®^ Acute Myeloid Leukemia Cancer Panel, IDT). We have previously used this panel for the identification of pre-leukemic mutations in peripheral blood leukocytes of individuals who later developed acute myeloid leukemia (15).

### Cell line dilution series

To evaluate analytical performance of mutational profiling, we created cell line dilution series using sheared genomic DNA from cancer cell lines with known genetic alterations to emulate varying levels of mutant allele frequencies (Supplementary Table S1). The source of cell line genomic DNA was as follows: MOLM13 was obtained from DSMZ, SW48 was obtained from ATCC, HCT116 was a kind gift of Dr Daniel De Carvalho, and MM1S was obtained from Dr Rodger Tiedemann. For LargeMid, we performed a dilution series at ratios of 1/5 in duplicate from 5% to 0.04% (six dilution points including 100% and 0% levels, *n* = 2 libraries per dilution point, total of 12 libraries). For SmallDeep, we used a dilution series at ratios of 1/10 from 1:1 to 1:10^6^ (eight dilution points including 100% and 0% levels, *n* = 1 library per dilution point, total of eight libraries).

### Next-generation sequencing library preparation

Illumina-compatible next-generation sequencing (NGS) libraries were prepared for each dilution point from genomic DNA. Briefly, 60–100 ng DNA was sheared before library construction using a Covaris M220 sonicator (Covaris, Woburn, MA, USA) to attain median fragment sizes of 180–250 bp. The DNA libraries were constructed using the KAPA Hyper Prep kit (#KK8504, Kapa Biosystems, Wilmington, MA, USA) with custom adapters containing 2 bp in-line duplex unique molecular identifiers (UMIs, Supplementary Tables S2 and S3). Following end repair and A-tailing, we performed adapter ligation overnight using 100-fold molar excess of adapters. Agencourt AMPure XP beads (Beckman-Coulter) were used for library clean up and ligated fragments were amplified between 4 and 8 cycles using 0.5 μM Illuminal universal and sample-specific index primers.

### Target capture and sequencing

Indexed Illumina libraries were pooled together in a single capture hybridization (Supplementary Table S1). Following the IDT Hybridization capture protocol, each pool of DNA was combined with 5 μl of 1 mg Cot-I DNA (Invitrogen) and 1 nmol each of xGen Universal Blocking Oligo (Integrated DNA Technologies, Coralville, IA, USA) to prevent cross hybridization and minimize off-target capture. Samples were dried and re-suspended in hybridization buffer and enhancer. Target capture with custom xGen Lockdown Probes (Integrated DNA Technologies, Coralville, IA, USA) was performed overnight. Streptavidin-coated magnetic beads were used to isolate hybridized targets according to manufacturer's specifications. Captured DNA fragments were amplified with 10–15 cycles of PCR. Pooled libraries were sequenced using 100–125 bp paired-end runs on Illumina platforms (HiSeq v3 2000, HiSeq 2500) at the Princess Margaret Genomics Centre (www.pmgenomics.ca). NGS libraries for SmallDeep and LargeMid were sequenced to an average of 186 312× and 4223× target coverage, respectively (see QC metrics in Supplementary Table S1).

### Data preprocessing

Sequencing reads were de-multiplexed using sample-specific indices followed by removal of the first 3 bp of each read, as these correspond to the 2 bp UMI and single T invariant spacer sequence necessitated for ligation. Reads without the invariant T sequence were discarded as they were not compliant with this design. The extracted UMIs from paired-end reads were grouped and written into the FASTQ sequence identifier header of each read for downstream *in silico* molecular identification. FASTQ files were mapped to the human reference genome hg19 using BWA (v 0.7.12) (16), processed using the Genome Analysis ToolKit (GATK) IndelRealigner (v 3.4-46) (17), and sorted by genome position and indexed using SAMtools (v 1.3) (18). This process created sorted BAM files containing sequence alignment data.

### Barcodes used in UMIs

Short oligonucleotide barcodes have the benefit of reduced cost for barcode synthesis and conservation of nucleotide bases for biological DNA in short read sequencing. To characterize unique molecules, we utilized a 4 bp barcode (comprised of a pair of 2 bp in-line UMIs on the end of each fragment) in combination with four sequence features from paired-end reads: (i) genomic position, (ii) concise idiosyncratic gapped alignment report (CIGAR), (iii) read orientation and (iv) read number. Hybridization capture approaches have the benefit of catching a wide range of molecules with varying mapping positions, whereas amplicon-based methods capture fragments with conserved positions. By utilizing the diverse genome mapping locations of hybrid capture fragments, shorter barcodes can be employed in combination for unique molecular identification (10).

### Analysis of single strand UMIs

Using our UMIs, reads derived from the same strand of a molecule were condensed into single strand consensus sequences (SSCS). First, a filter was applied to exclude reads which were unmapped, paired with an unmapped mate, or had multiple alignments. Paired reads were assigned UMIs as described above using barcode, genome mapping, CIGAR string, strand of origin, orientation, and read number information. Reads sharing the same UMIs were grouped into the same read family. Only families with 2 or more members were error suppressed and collapsed to form SSCSs as following:

- For each position across a sequence length, a Phred quality threshold of Q30 was enforced for every read (only bases with an error probability of one in a thousand or less (>Q30) were evaluated for consensus formation).
- The most frequent base at each position across all replicate reads of the same molecule was established as the consensus. The most common base was assigned if the proportion of reads representing that base was greater than or equal to the threshold required to confidently call a consensus (default cutoff 0.7—based on previous literature (9)), otherwise an *N* was assigned.
- As each SSCS represents multiple reads derived from the same strand of a unique fragment, a consensus query name was assigned to each SSCS pair. Similar to our UMIs, the pairing tag consists of a barcode along with four sequence features: (i) genome mapping ordered by coordinate, (ii) strand of origin inferred from read orientation and number, (iii) CIGAR string ordered by strand of origin and read number and (iv) read family size (number of reads supporting SSCS).

### Singleton correction

We developed two approaches for Singleton Correction using the duplex nature of DNA molecules for elimination of technical artefacts. Following the formation of SSCS, singletons were grouped with their complementary SSCS for (i) Singleton Correction by SSCS. If a complementary SSCS could not be identified, single reads were paired with their complementary singleton for (ii) Singleton Correction by singletons. Through this step-wise approach, reads corresponding to the dual strands of a template molecule were used to perform Singleton Correction as following:

- UMIs were assigned to singleton and SSCS reads. For each singleton, a duplex identifier was determined by interchanging barcodes and switching the read number. If R_1_ and R_2_ on a positive strand had AC/GT as barcodes, their duplex barcodes would be GT/AC on the minus strand. R_1_ in the forward orientation on the plus strand corresponds to R_2_ in the forward orientation on the minus strand.
- Singleton Correction was achieved using either a complementary (i) SSCS or (ii) singleton corresponding to the opposite DNA strand. For each base, a Phred quality filter of Q30 was enforced to remove error prone bases. Consensus sequences were established by taking concordant bases at each position and assigning *N*s for mismatches.
- Error suppressed singleton pairs were assigned a consensus query name as described above for SSCS reads.

Recovered singleton were written to separate BAM files depending on method of correction (i.e. Singleton Correction by SSCS or Singleton Correction by singletons). They were subsequently merged with SSCS reads for downstream duplex formation.

### Analysis of duplex barcodes

For optimal error suppression, duplex consensus sequences (DCS) can be established by condensing SSCSs that originated from opposite/complementary strands of a template DNA molecule. This second layer of duplex error suppression eliminates asymmetric strand artefacts. DCSs were established by preserving matched bases between reads from complementary strands. Although DCSs have the lowest rates of error, they only depict a portion of the total molecular population. To portray accurate molecular representation for variant calling, a BAM file containing all unique molecules was created by combining DCS, SSCS (without duplex pair), and uncorrected singletons.

### Error analysis

We determined base substitution (error) rates using the integrated digital error suppression (iDES) tool (https://cappseq.stanford.edu/ides/download.php#bgReport) (10). BAM files were first converted to base frequency files for each genomic position using *ides-bam2freq.pl*. With the *ides-bgreport.pl*, background errors were calculated using non-reference bases <5% allele frequency with at least one read support. Error rates were determined as the number of non-reference bases over all sequenced bases within our targeted panel. We evaluated error rates at each step of error correction.

### Recovery efficiency

Efficiency of consensus formation reflects the frequency of consensus sequences generated per read. This is determined by the average number of reads needed to construct a consensus sequence. For example, an efficiency rate of 10% indicates each read contributes to 0.1 of a consensus sequence, or 10 reads are needed to form a single consensus sequence.

In order to compare targeted panels of different sizes, efficiency rates were calculated using the mean target coverage (cov). GATK (v 3.6) DepthOfCoverage was used to determine mean fragment coverage per target position. Notably, we performed fragment counting as it considers overlapping reads as a single entity rather than double-counting those reads:

\begin{equation*}{ Efficiency}\ = \ \frac{{ cov}_{\left ( { DCS\ or\ SSCS} \right )}}{{ cov}_{{ uncollapsed}}}\end{equation*}

As DCS formation is dependent on the number of SSCS and corrected singletons, DCS recovery rates were estimated by comparing observed over expected rates:

\begin{equation*}\ { Recovery}_{{ DCS}} = \frac{{{ observed}_{{ DCS}}}}{{{ expected}_{{ DCS}}}}\ = \frac{{{ cov}_{{ DCS}}}}{{\left( {\frac{{{ Cov}_{{ SSCS}}}}{2}} \right)}}\ \end{equation*}

### Comparison of previous UMI methods

Error rates (Supplementary Figure S1) and efficiency rates (Figure 1B) of previous methods were obtained as follows: Schmitt *et al.* error rates were reported in the text and efficiency rates were derived from Supplementary Table S1 (8), Kennedy *et al.* efficiency rates were obtained from Supplementary Table S1 (9), Schmitt *et al.* error rate and DCS efficiency rate (duplex nucleotides/(reads × (101 – 17)) were derived from Supplementary Table S2 (19), Newman *et al.* error rates were reported in text and efficiency rates were approximated from Supplementary Figure S8 (10). Efficiency rates were calculated with the equation described under ‘Recovery efficiency’, unless otherwise specified.

### *In silico* downsampling

To compare hybrid capture panels of different sizes sequenced to various depths, we performed downsampling of sequencing coverage for each sample. We chose nine intervals ranging between 128 000× and 500× coverage. Each sample was reduced to the set intervals through *in silico* downsampling of paired-end reads with Samtools (v 1.3). This process was repeated 10 times for each sample across all intervals to address sampling biases. Each downsampled file was then processed through our barcoding pipeline to generate individual BAM files for singletons, SSCS, DCS, Singleton Correction by SSCS, and Singleton Correction by singletons. Corrected singletons were merged with SSCS to generate SSCS (SC) for downstream formation of DCS (SC). Efficiency for consensus formation and molecular recovery was assessed for each error suppression strategy across the broad range of coverage intervals.

### Cell line dilution mutation analysis

To assess the sensitivity and specificity of UMI-based error suppression utilizing Singleton Correction, we analyzed mixed cancer cell lines diluted in 1/5 fractions across two technical replicates (Supplementary Table S4). We focused our analysis on the LargeMid library to evaluate the impact of Singleton Correction as the ultra-deeply sequenced SmallDeep contained very few singletons. For sensitivity, we evaluated single nucleotide polymorphisms corresponding to the MOLM13 cell line spiked into the dilution series. We curated a list of heterozygous (40–60% AF) and homozygous (>95% AF) single nucleotide polymorphisms (SNPs) overlapping the targeted panel that were not present in the background cell line above 1% AF. There were 222 SNPs common between technical replicates with four SNPs identified only in one of the replicates as a result of our AF thresholds. Variant calls were generated for each sample using Varscan2 (v. 2.4.2) (20). We calculated sensitivity using the list of candidate SNPs across uncollapsed and consensus reads. When assessing specificity, we bootstrapped 222 positions across the 1.2 MB targeted panel excluding sites with potential variants from both cell lines. To prevent inflation of errors, we excluded regions with poor alignability scores (obtained from ENCODE). We enumerated false positives within randomly sampled positions across 1000 iterations to evaluate specificity.

### Analysis of patient samples

In our analysis, we selected samples reported to have putative driver mutations of acute myeloid leukemia (AML) (Abelson *et al.*Supplementary Table S2.1) and healthy age- and sex matched controls. We obtained 291 BAM files of peripheral blood leukocyte samples from Abelson *et al.* (15). In addition, we received 10 BAM files of umbilical cord blood samples with hybrid capture using the same 1.2 Mb leukemia panel (xGen^®^ Acute Myeloid Leukemia Cancer Panel, IDT) sequenced to similar depths as the peripheral blood samples. UMIs were previously extracted and appended to the query name of each file. The BAM files were aligned with BWA mem to the Genome Reference Consortium Human build 37 (GRCh37).

The 10 umbilical cord blood samples were obtained from Trillium Hospital (Mississauga, Ontario, Canada) with informed consent in accordance to guidelines approved by the University Health Network Research Ethics Board. Cord blood was processed 24–48 h post-delivery. Mononuclear cells were enriched using Ficoll-Paque followed by red blood cells lysis by ammonium chloride and CD34+ selection prior to DNA extraction. 100 ng genomic DNA from the umbilical cord blood samples was used for library preparation and target capture sequencing as described above.

We processed the reads using our duplex UMI method with or without Singleton Correction. We carried out consensus efficiency and error rate as described above. To assess variant detection performance, we used 391 pre-leukemic mutations reported by Abelson *et al.* as a gold standard list (Supplementary Table S2.1, excluding one mutation that was not present in our BAM files). Files were analyzed to detect single nucleotide variants (SNVs) and small indels using Varscan2 (20). We calculated sensitivity using the 391 pre-leukemic mutations and the 224 samples (67 samples from pre-AML individuals and 157 samples from age- and sex-matched controls) in which they were reported. Additionally, we assessed specificity using the 391 pre-leukemic mutations in all 301 samples, excluding reported mutations from Abelson *et al.* Specificity was similar when only considering a subset (77) of the 301 samples, including the 10 umbilical cord blood samples and 67 control samples not found to have pre-leukemic mutations by Abelson *et al.* (Supplementary Table S5).

## RESULTS

### Low efficiency consensus sequence assembly with traditional UMI methods

To assess the potential impact of Singleton Correction across diverse datasets, we first calculated important metrics of consensus sequence assembly from prior landmark studies that used traditional UMI methods (Figure 1) (8–10). This revealed critical inefficiencies in constructing SSCSs (efficiency ≤ 25%) and DCSs (efficiency ≤ 2.5%) when singletons are excluded. To confirm this using newly generated data, we performed hybrid capture NGS on cancer cell line genomic DNA with either a large panel sequenced to moderately deep coverage (LargeMid; 1.2 Mb panel, 4223× average depth) or a small panel sequenced to ultra-deep coverage (SmallDeep; 13 kb panel, 186 312× average depth). With two or more redundant reads required to construct a consensus sequence, only two-thirds of all reads in LargeMid qualified for traditional error suppression; this corresponded to a 25% SSCS efficiency rate and 2% DCS efficiency rate (Figure 1B). Since two SSCSs are required to form a DCS, theoretically we expect the maximum frequency of DCS recovery to be half of total SSCSs. However, only 15% of the expected DCSs were observed in LargeMid, and the more deeply sequenced libraries had only modest gains in DCS recovery (SmallDeep and (8–10)).

### Singleton Correction augments consensus sequence assembly efficiency

We reasoned that the low consensus efficiency and DCS recovery rates observed with traditional UMI methods could be attributed to the high rate of singletons. Indeed, when Singleton Correction was applied to the LargeMid dataset, efficiency increased to 33% for SSCS and 9% for DCS. This improvement in efficiency of consensus sequence assembly resulted in a 3.6-fold increase in DCS recovery (53%) compared to traditional duplex UMI methods. In contrast to the LargeMid dataset, the vast majority (98.7%) of reads in the SmallDeep dataset contributed to consensus sequences. With so few singletons available in SmallDeep, Singleton Correction had a negligible impact on SSCS and DCS formation (Figure 1B).

### High quality error suppression using singletons

**Figure 2.**
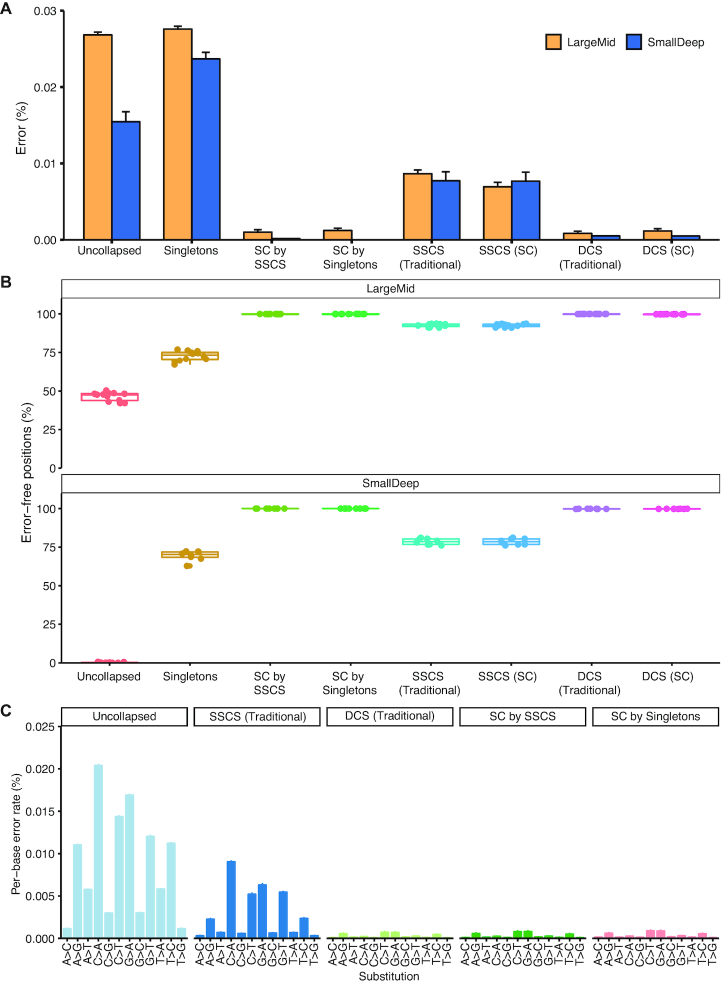
Corrected singletons have error profiles similar to high-quality Duplex Consensus Sequences. Comparisons between (**A**) selector-wide error rates and (**B**) error-free positions in the LargeMid (*n* = 12 libraries) and SmallDeep (*n* = 8 libraries) cell line datasets. Low rates of error and high frequency of error-free positions in Singleton Correction (SC) of SmallDeep may be attributed to the low presence of singletons within the sample and even fewer singletons corrected, as it had ultra-deep sequencing. As such, we focused our analysis on the LargeMid cell line dataset to assess (**C**) per-base error rates across different base substitutions to evaluate the error profile within corrected singletons (SC by SSCS and SC by Singletons). Data are presented as mean ± S.D.

We next evaluated the quality of the singletons that participated in Singleton Correction to assess their suitability for error suppression. Singleton Correction reduced the per-base error rate of singletons by 25-fold from 0.028% to 0.0011% (Figure 2A). Error rates in DCSs augmented by Singleton Correction were comparable to traditional DCSs in our datasets (Figure 2 and Supplementary Figure S3) and those from previous reports (8–10,19) (Supplementary Figure S1). This suggests high quality error suppression can be achieved using singletons, challenging the fundamental notion of requiring redundant reads for correction in traditional UMI-based methods.

### Influence of sequencing depth on the impact of Singleton Correction

**Figure 3.**
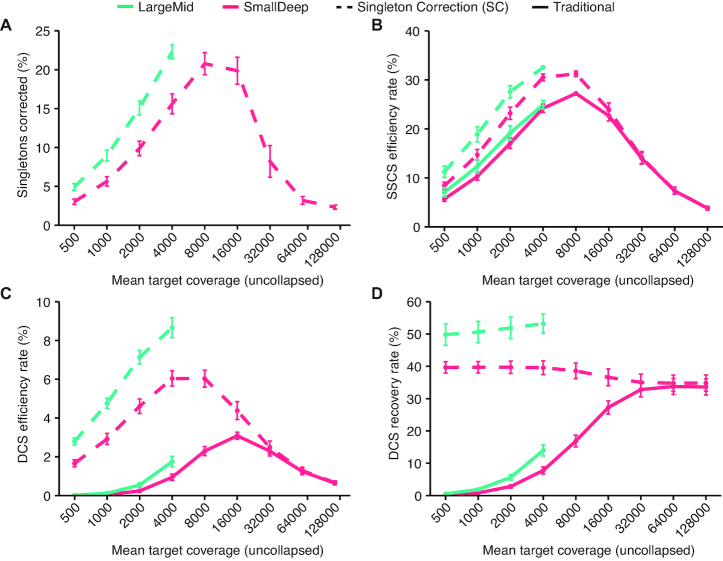
Singleton Correction impacts consensus formation and variant calling performance. (**A–D**) A traditional UMI approach is compared with Singleton Correction across nine coverage intervals. Plots are shown for LargeMid (*n* = 12 libraries) and SmallDeep (*n* = 8 libraries) cell line datasets that were downsampled from 128 000× to 500× depth, decreasing by half at each interval, across ten simulations. Mean target coverage is log_10_ transformed to show trends at lower range. Uncollapsed reads are defined as unprocessed reads that have not been collapsed into consensus sequences. See Methods for efficiency and recovery rate calculations.

Since we observed a much greater effect of Singleton Correction on consensus efficiency and DCS recovery in the LargeMid dataset compared with the SmallDeep dataset, next we formally assessed the influence of sequencing depth on the impact of Singleton Correction. We performed downsampling of SmallDeep and LargeMid sequencing reads to achieve sequencing depths between 500× and 128 000× and then applied consensus assembly with or without Singleton Correction. Both SmallDeep and LargeMid displayed similar trends in consensus efficiency and recovery with a greater proportion of singletons corrected as sequencing depth increased (Figure 3A–D). Peak Singleton Correction rate occurred at 8000× depth, where 21% of singletons were corrected. This high rate was nearly maintained up to 16 000×, but at ≥32 000× a smaller proportion of singletons underwent Singleton Correction, suggesting an increased prevalence of duplicate reads. Analysis of SSCSs revealed consistent trends, with decreased efficiency beyond 8000× depth, indicating saturation of unique molecules with duplicate reads (Figure 3B). While Singleton Correction contributed only minor improvements to SSCS efficiency, DCS efficiency improved >2-fold at sequencing depths where singletons were abundant (Figure 3C). Furthermore, Singleton Correction enhanced DCS recovery at every coverage interval we sampled (Figure 3D). Thus, Singleton Correction ameliorated the inefficiencies of traditional UMI methods and achieved optimal recovery of DCSs across a wide range of sequencing depths. The overall impact of Singleton Correction was muted at ≥32 000× depth due to saturation of unique molecules in the dataset.

### Increasing sensitivity with Singleton Correction

**Figure 4.**
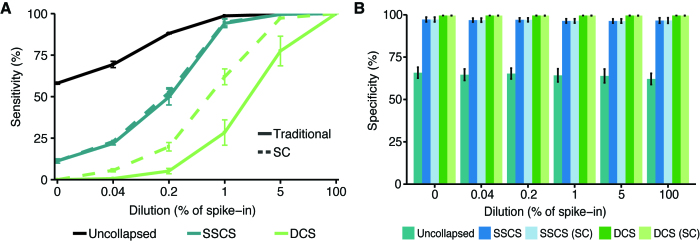
Variant detection sensitivity is improved with Singleton Correction. (**A**) Serial dilutions of cell lines MOLM13 spiked into SW48 (LargeMid, *n* = 12, 2 replicates per dilution). Sensitivity was assessed using 222 single nucleotide polymorphisms (SNPs) unique to the spike-in cell line that were not found in the background cell line at allele frequencies >1%. (**B**) Specificity was evaluated through bootstrapping 222 positions across the targeted panel (1000 iterations); regions of potential variants in either cell line were excluded from sampling. Data are presented as mean ± S.D.

Next, we compared the detection of 222 high-confidence germline variants from the MOLM13 cell line not found in SW48 (LargeMid dataset) using duplex UMI methods with and without Singleton Correction (Supplementary Figure S4A). Using mixed cancer cell lines, we emulated varying levels of mutation variant allele frequencies at 5-fold dilutions from 100% to 0.04% MOLM13 (Supplementary Figure S4B). Across all the dilutions, uncollapsed reads had the highest sensitivity (58–100%) and the lowest specificity (62–66%). Likewise, SSCSs displayed greater sensitivity than DCSs at the expense of reduced specificity (∼97%). Although the inclusion of Singleton Correction resulted in minimal difference for SSCS, DCS sensitivity increased on average by 18% without a detriment in specificity (∼99.5%). At 0.04% MOLM13, the lowest dilution point, Singleton Correction produced an 8-fold increase in DCS sensitivity from 0.68% to 5.63% (Figure 4A, B). These results demonstrate the potential of Singleton Correction for high-confidence detection of low-frequency variants.

### Validation of Singleton Correction performance in clinical samples

**Figure 5.**
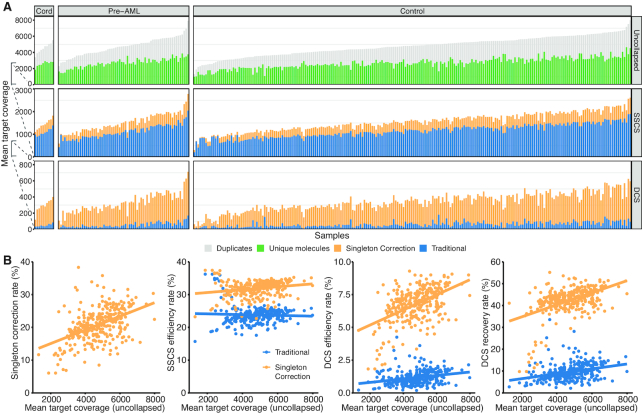
Performance of Singleton Correction in targeted sequencing of 301 peripheral blood leukocytes. (**A**) Plot is divided into columns corresponding to the three sample types (umbilical cord blood, blood from healthy volunteers, and blood from pre-leukemia individuals) and further separated into rows with each panel indicating the proportion of depth corresponding to uncollapsed, SSCS, and DCS reads. Each panel contrasts the reads derived from a traditional UMI strategy with reads from Singleton Correction. (**B**) Scatter plots of mean target coverage from uncollapsed reads versus Singleton Correction rate, SSCS efficiency rate, DCS efficiency rate, or DCS recovery rates; a traditional UMI approach is compared with Singleton Correction. See Methods for efficiency and recovery rate calculations and interpretations.

To investigate the impact of Singleton Correction in clinical samples, we next applied our method to a large study on pre-leukemia mutation detection from peripheral blood (15). Peripheral blood genomic DNA samples from 301 individuals were sequenced using the 1.2 Mb LargeMid panel to an average depth of 4746× (Figure 5A). This cohort consisted of 67 pre-leukemia patients and 224 age- and sex- matched individuals (controls) (15) as well as 10 umbilical cord blood samples that served as additional controls. Across the entire cohort, over half of all sequenced reads were unique molecules (singletons) with the remainder comprised of duplicate reads. With a traditional UMI correction method, the efficiency rate was on average 24% for SSCSs and 1% for DCSs (Figure 5B). Singleton Correction increased efficiency by 8% in SSCS and 6% in DCS and increased duplex recovery by 4-fold from 9.6% to 42%. We again observed a positive correlation between Singleton Correction and sequencing depth (Supplementary Figure S5A). Furthermore, we found consistent efficiency rates with the LargeMid cell line dilution experiment that employed a similar sequencing depth.

**Figure 6.**
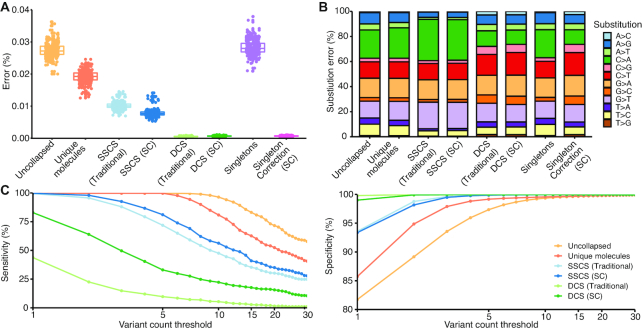
Error suppression and detection of low-frequency variants in clinical samples. (**A, B**) Selector-wide error rates and substitution profiles across reads with varying levels of error correction. Consensus sequences from a traditional UMI approach are compared with those derived from Singleton Correction. (**C**) Sensitivity and specificity of SNV calls at variant count thresholds from 1 to 30 for 391 putative driver mutations of acute myeloid leukaemia from the original study by Abelson *et al.* (15). Sensitivity was assessed in 224 samples in which the 391 mutations were reported. Additionally, we assessed specificity using the 391 mutations in all 301 samples, excluding exact matches from Abelson *et al.*

Singleton Correction expanded the number of reads corrected without inflating the overall error rate in patient samples. With a traditional UMI correction method, error rates were 0.01% in SSCSs and 0.0005% in DCSs. Our method reduced the error rate within singletons to 0.0007%, which was comparable to the DCS error profile (Figure 6A) and to the cell line findings (Figure 2). Error substitution profiles reflected a signature of oxidative damage in reads without duplex correction (21). Notably, the characteristic imbalance between G>T and C>A substitutions was eliminated in singletons that underwent Singleton Correction (Figure 6B, Supplementary Figure S5B). These results validate our findings from cell lines and indicate that Singleton Correction is a generalizable approach that can improve the performance of UMI-based techniques in clinical samples.

### Detection of low-allele-frequency variants in clinical samples

We next evaluated the effect of Singleton Correction on mutation detection accuracy in this cohort of clinical samples. Using 391 putative driver mutations of AML from Abelson *et al.* (Supplementary Figure S6A), we assessed sensitivity and specificity of duplex UMI methods. Within the different consensus data types, we evaluated performance across a range of variant count thresholds between 1 and 30; variant counts were used as opposed to variant allele fractions because of the skewed (overestimated) distribution of variant allele fractions often present within consensus sequences (Supplementary Figure S6B). Of the consensus data types, the aggregate of all unique molecules (i.e. merged DCSs, SSCSs and singletons) had the highest sensitivity but also low specificity due to inclusion of uncorrected singletons (Figure 6C). While traditional DCS had near perfect specificity without any additional filtering, sensitivity was less than half of SSCS. Singleton Correction improved sensitivity of DCS by 39% while maintaining specificity >99%.

## DISCUSSION

The ability to detect low-allele-frequency variants with high-throughput sequencing technologies is dictated by the quantity of template DNA molecules, sequencing depth, and level of technical artefacts. Effective error suppression strategies are needed as errors determine the threshold at which true genetic variants can be discerned from false positives. False positive mutation calls are particularly problematic when the analysis space spans many thousands of bases, as is the case for some commercial sequencing services (32–34). Methods reported to date have not been capable of achieving high accuracy mutation detection at low thresholds without ultra-deep sequencing and/or sacrificing template DNA molecules, or without the use of large control cohorts for modeling background error rates (10). In this study, we present an enhanced UMI-based error correction methodology aimed at addressing these important limitations.

Traditional UMI-based error correction methods require deep sequencing to achieve multiple redundant reads from the same template DNA molecule. For instance, Duplex Sequencing creates high quality DCSs with exceedingly low error rates but at the expense of inefficient processes leading to critical losses of template DNA molecules (14,15,18). Here, we demonstrate that Singleton Correction is a powerful extension for UMI-based error correction because it enables high quality error suppression across a greater number of reads. Indeed, through Singleton Correction we found that the benefits of duplex UMI methods can be extended to singletons, and therefore these reads no longer need to be categorically excluded from error suppression procedures (8–10,22). As a result, Singleton Correction results in higher consensus sequence efficiency and recovery compared to traditional methods.

Singleton Correction can be incorporated into any duplex UMI method (6,8–10,19). We used custom duplex UMI-containing adapters and sequenced on an Illumina platform, but other commercial and custom implementations of duplex UMIs for Illumina and alternative sequencing platforms would also benefit by incorporating Singleton Correction. We found the greatest benefit in hybrid capture NGS datasets with sequencing depths ≤16 000×. Amplicon NGS datasets would be expected to benefit less, since they generally contain fewer singletons compared with hybrid capture NGS.

Despite the gains in DCS recovery achieved using Singleton Correction compared with traditional UMI methods, still 40–50% of expected DCSs were not recovered. This could be explained by losses that are known to occur during upstream library preparation and sequencing (23), which cannot be completely overcome through over-sequencing or Singleton Correction. Further innovations in library preparation and/or sequencing methodologies may be required to realize even greater improvements in DCS recovery.

Based on our data, an important benefit of incorporating Singleton Correction is an increase in sensitivity for detecting low-frequency variants without compromising specificity. We confirmed this result using both a cell line dilution series as well as a large cohort of clinical samples that included individuals with pre-AML and/or age-related clonal hematopoiesis. High specificity is particularly important for noninvasive genotyping or screening applications (24), for instance in the setting of early detection of AML in otherwise healthy individuals (15), as false positive results may lead to unnecessary procedures and distress. Taken together, our results will inform future prospective studies in which NGS is conducted on peripheral blood or circulating DNA for early cancer detection and for other applications in oncology and precision medicine.

## DATA AVAILABILITY

The dataset generated and analyzed during the current study are available in the NCBI Sequence Read Archive (SRA; https://www.ncbi.nlm.nih.gov/sra/) under access numbers SRP140497 and SRP141184. Software is available as supplementary material and on GitHub under https://github.com/pughlab/ConsensusCruncher.

## Supplementary Material

gkz474_Supplemental_Files

## Notes

Present address: Scott V. Bratman, MaRS Centre, 101 College Street, Princess Margaret Cancer Research Tower, Room 13-305. Princess Margaret Cancer Centre, University Health Network, Toronto, ON M5G 1L7, Canada.

Present address: Trevor J. Pugh, MaRS Centre, 101 College Street, Princess Margaret Cancer Research Tower, Room 9-305. Princess Margaret Cancer Centre, University Health Network, Toronto, ON M5G 1L7, Canada.
