# Supplementary material for: High efficiency error suppression for accurate detection of low-frequency variants
Source: Nucleic Acids Res. 2019 May 25;47(15):e87. doi: 10.1093/nar/gkz474 (PMC6735726; doi:10.1093/nar/gkz474)
Supplement: gkz474_Supplemental_Files [file gkz474_supplemental_files.zip › Wang_Supplementary_Information_2019-04-28.pdf]

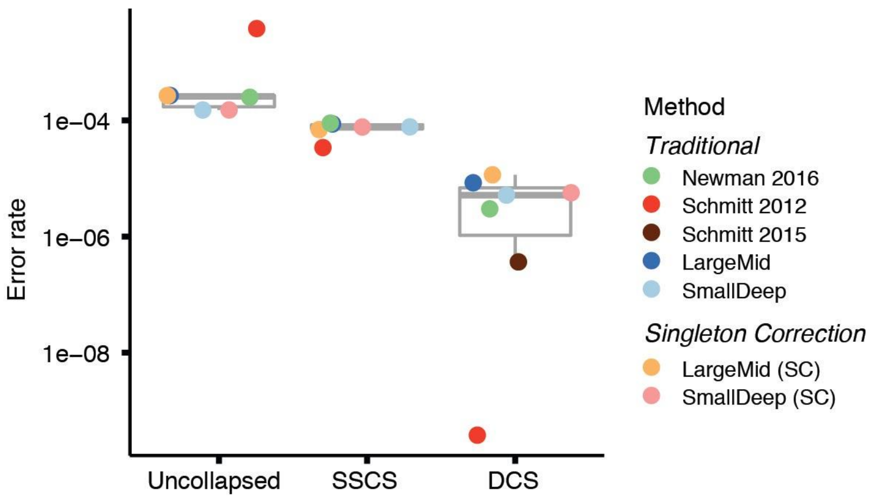

Supplementary Figure 1 | Singleton Correction achieves comparable error rates to traditional duplex UMI methods. Per-base error rates of traditional duplex UMI methods and Singleton Correction. We implemented both a traditional and Singleton Correction approach for the sequenced cell line genomic DNA in the LargeMid (n=12 libraries) and SmallDeep (n=8 libraries) cell line datasets in order to evaluate differences in error rate. Uncollapsed reads are defined as unprocessed reads that have not been assembled into consensus sequences. Corrected singletons were combined with single-strand consensus sequences (SSCS) and were subsequently formed into duplex consensus sequences (DCS) in Singleton Correction.

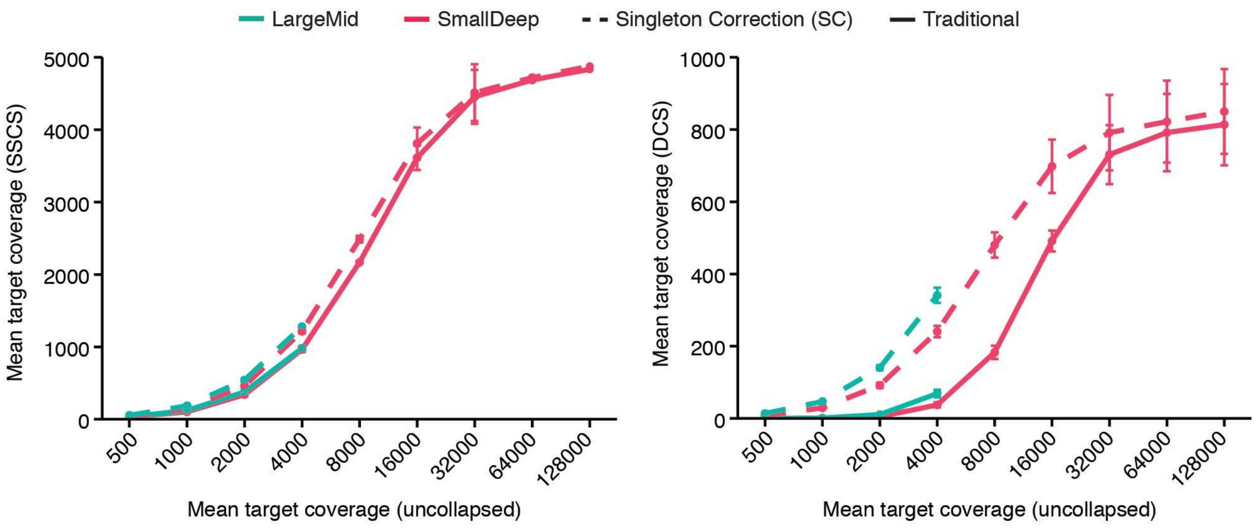

Supplementary Figure 2 | Consensus sequence formation is dependent on sequencing coverage. SmallDeep (n=8 libraries) and LargeMid (n=12 libraries) cell line datasets were downsampled to 9 sequencing depths ranging from 500x to 128,000x mean target coverage with 10 *in silico* replicates. Singleton Correction was compared against a traditional duplex UMI approach. Singleton Correction increased the mean collapsed target coverage as compared to traditional SSCS and DCS. Mean target coverage (x-axis) is log-scaled to show changes at lower depths. Data are presented as mean ± s.d.

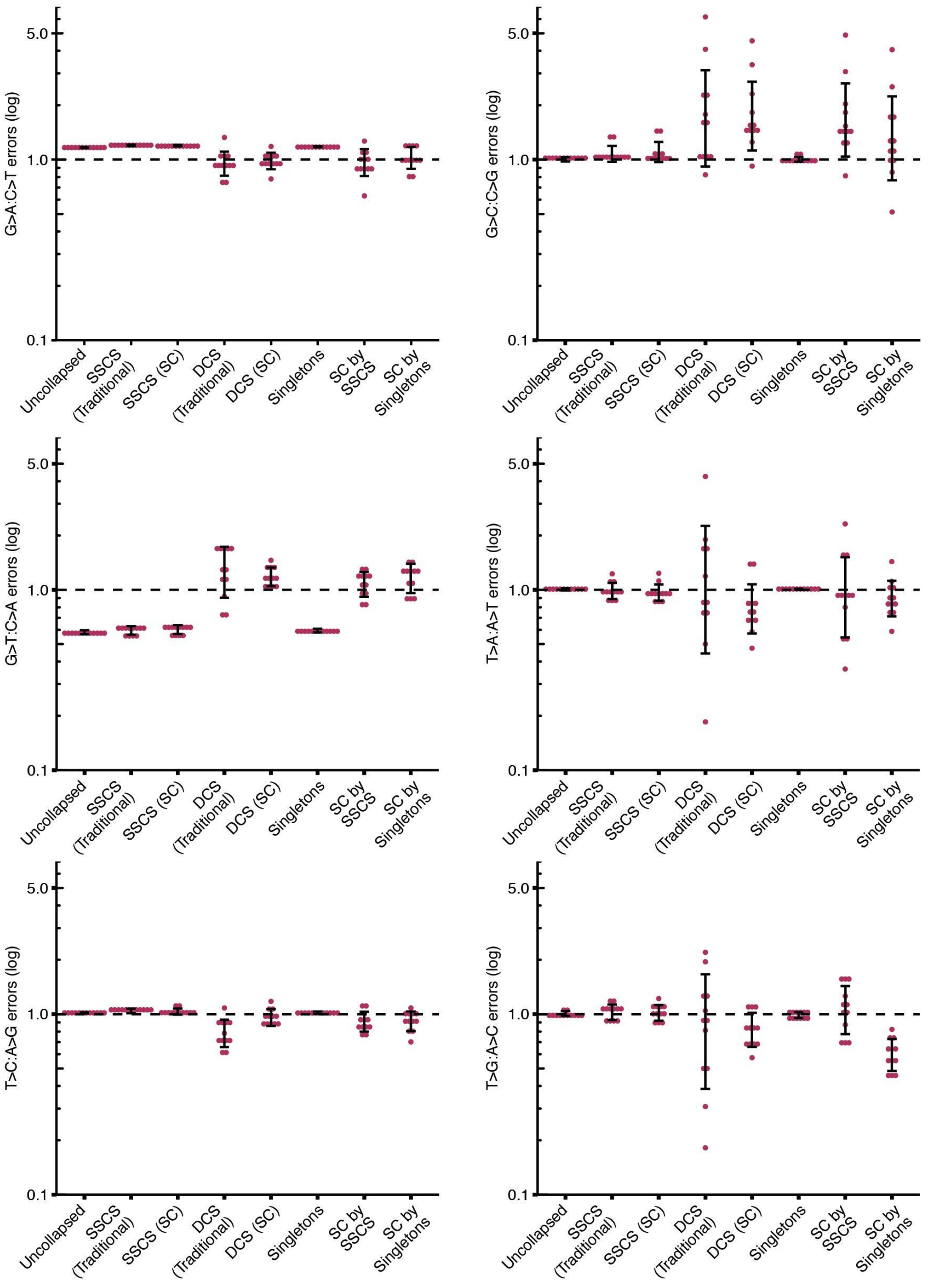
G>A:C>T errors (log)
G>C:C>G errors (log)
G>T:C>A errors (log)
T>A:A>T errors (log)
T>C:A>G errors (log)
T>G:A>C errors (log)
5.0
1.0
0.1
Uncollapsed
SSCS (Traditional)
SSCS (SC)
DCS (Traditional)
DCS (SC)
Singletons
SC by SSCS
SC by Singletons

Supplementary Figure 3 | Duplex strategies of error correction suppresses oxidative damage. Oxidation induced 8-oxoguanine converts to thymine after PCR amplification and arises as asymmetric strand lesions in DNA. As only plus strands are captured with our target panel comprised of negative probes, there is a bias for C>A errors. We evaluated 12 substitution classes across different consensus data types from the LargeMid cell line dataset (n=12 libraries) and compared ratio of errors between reciprocal base substitutions. Imbalances between G>T:C>A is indicative of oxidative damage.

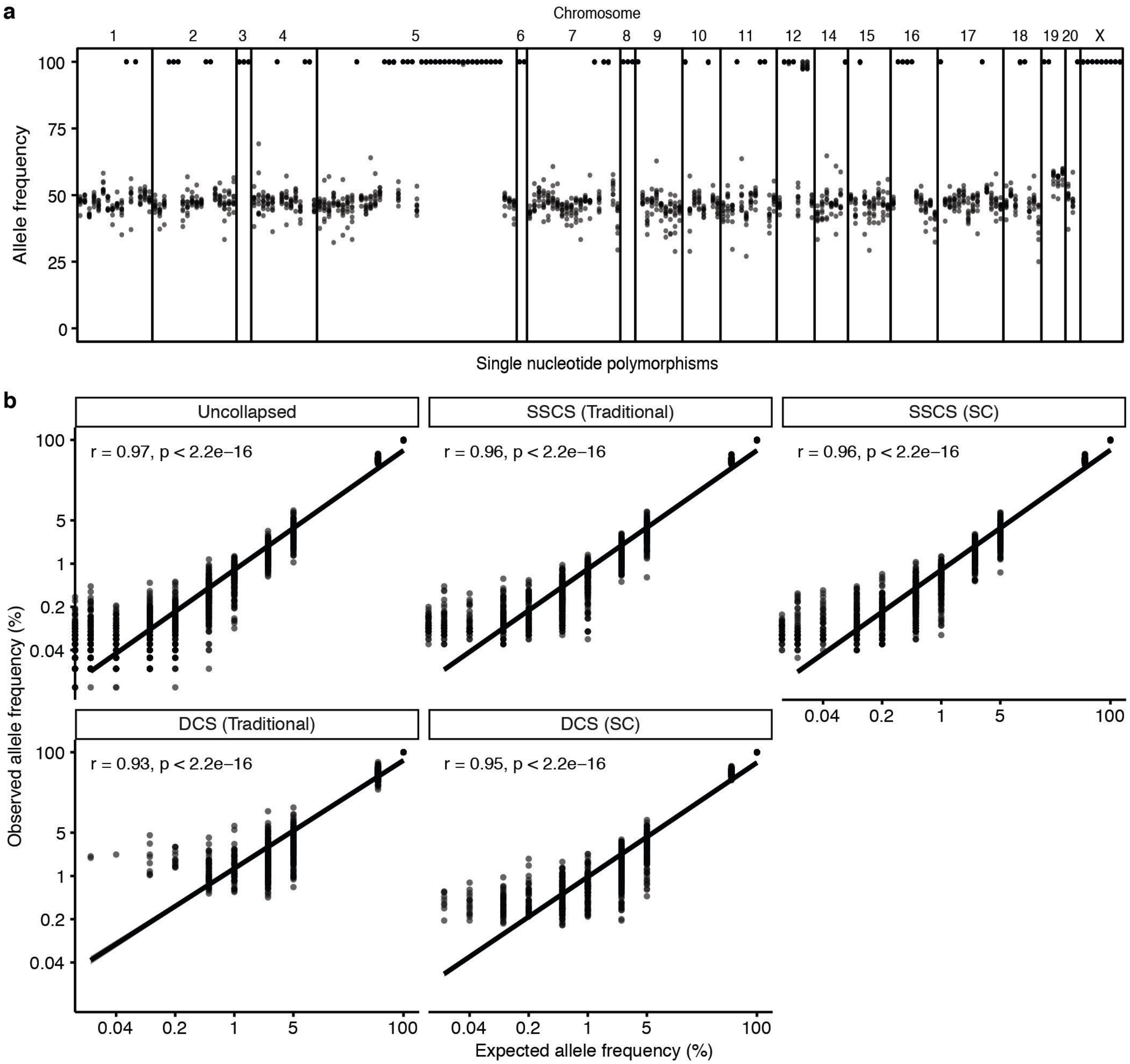

Supplementary Figure 4 | Mixed cancer cell lines emulate varying levels of mutant allele frequencies. a, 222 single nucleotide polymorphisms (SNPs) unique to the MOLM13 cell line, separated by chromosome. Allele frequency shown for the 100% undiluted cell line without any consensus processing (uncollapsed reads). **b**, Pearson correlation of observed and expected allele frequencies of 222 SNPs across uncollapsed and consensus sequences.

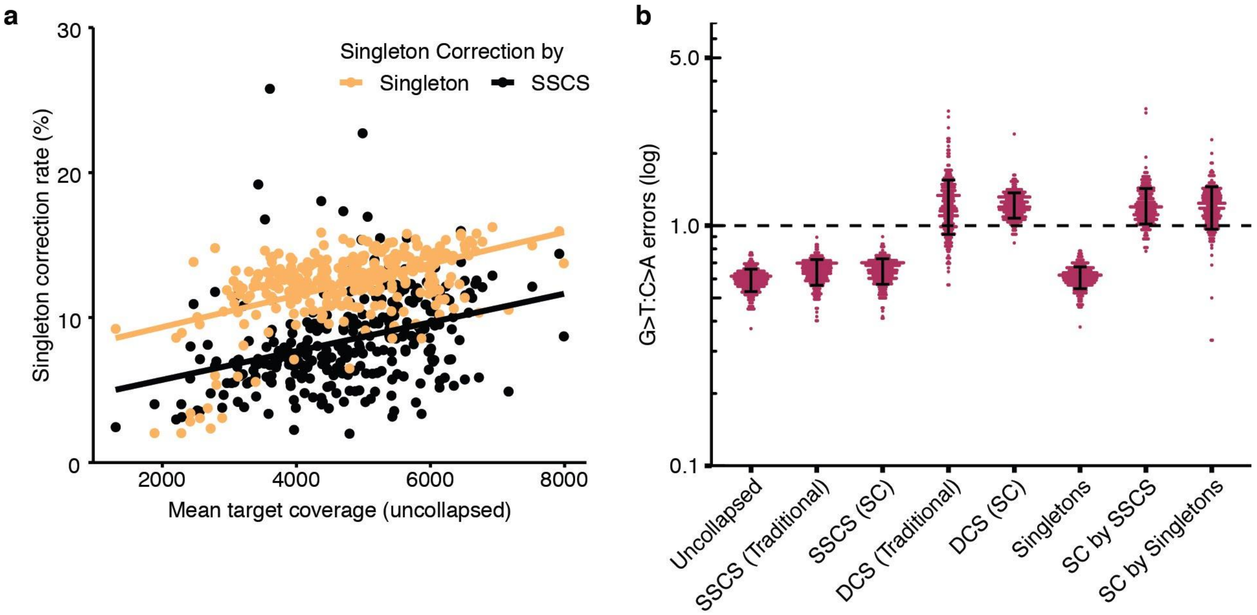

Supplementary Figure 5 | The performance of Singleton Correction in 301 clinical samples is consistent with the cell line dataset. a, Performance of Singleton Correction split by correction type (SSCS vs Singleton) across mean target coverage. Similar to the downsampled cell line data, we observe a positive correlation between the Singleton Correction rate among singletons and uncollapsed mean target coverage (Pearson, r=0.34, p<2.2e-16). Other singletons contribute more to Singleton Correction than SSCSs within these 301 samples. **b**, Oxidative damage characterized by an imbalance in substitution classes G>T:C>A is present in uncollapsed, SSCS, and all singletons but is suppressed in duplex corrected molecules (DCS and Singleton Correction).

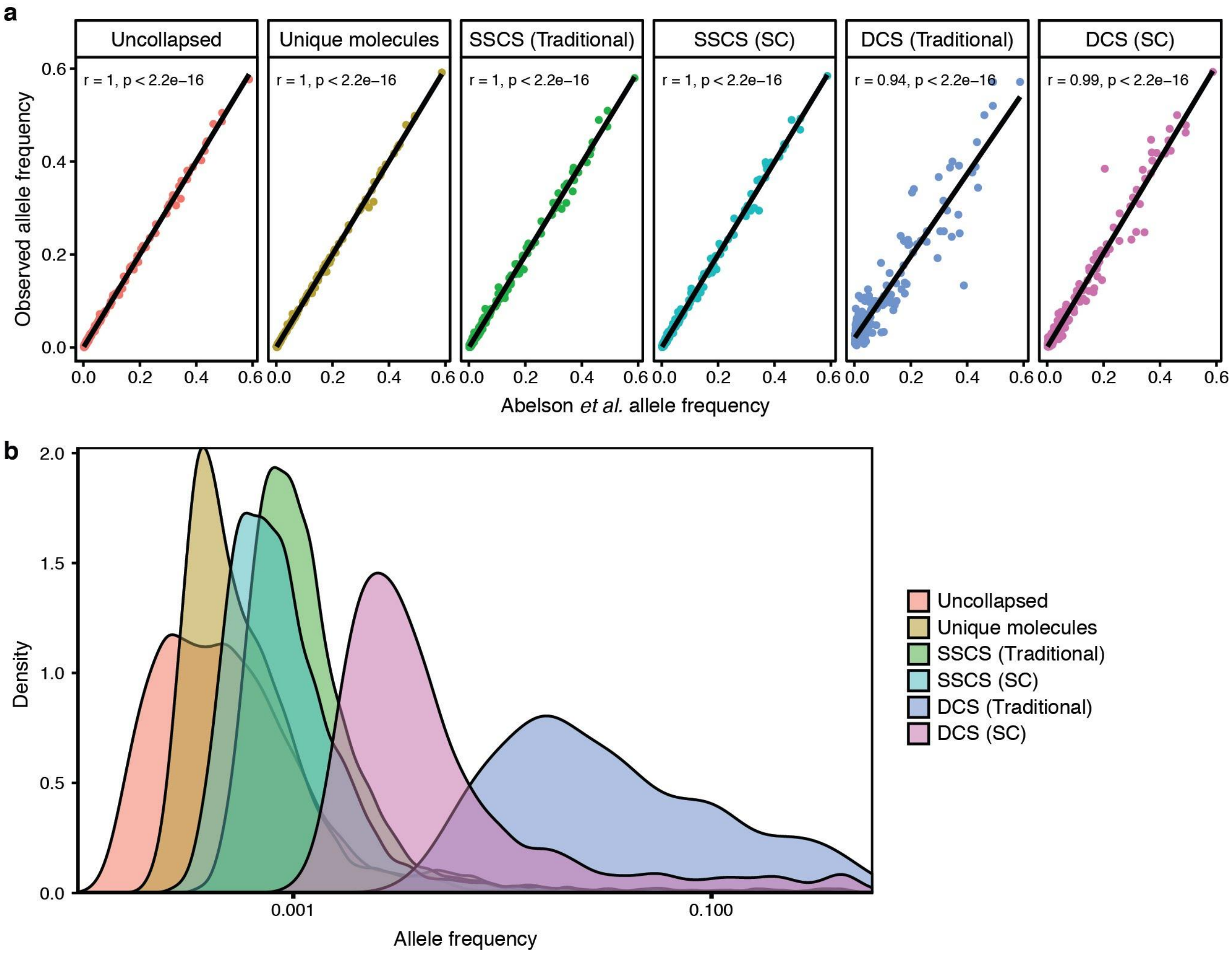

Supplementary Figure 6 | Allele frequencies in consensus data are skewed relative to uncollapsed. a, Allele frequency of 391 putative driver mutations of acute myeloid leukemia across uncollapsed and consensus data types. These observed allele frequencies are compared to reported values in Supplementary Table 2.1 from Abelson *et al* (n=224). **b,** Distributions correspond to the allele frequency of variants overlapping the 391 putative driver mutations, generated from different consensus data types of 301 samples. To show the effect of Singleton Correction on allele frequency, we assessed SSCS and DCS with a traditional correction approach and our methodology.

Project	LargeMid	SmallDeep
**Cell line spike-in**	MOLM13	HCT116
**Cell line background**	SW48	MM1S
**Dilution points**	6 (x2 technical replicates)	8
**Dilution (%)**	100, 5, 1, 0.2, 0.04, 0	100, 10, 1, 0.1, 0.01, 0.001, 0.0001, 0
**DNA input (ng)**	100	60
**Fragment size (bp)**	250	180
**Pre-capture PCR cycles**	8	4
**Samples pooled per capture**	3	4
**Target Panel**	xGen® AML Cancer Panel v1.0	All exons of BRAF, EGFR, KRAS, NRAS, PIK3CA
**Panel size (MB)**	1.2	0.01
**Post-capture PCR cycles**	10	15
**Sequencer**	HiSeq 2500	HiSeq 2000 v3
**Sequence length (bp)**	125	100
**Number of reads (billions)**	5.32	2.05
**Mean Target Coverage**	4,223	186,312
**On/Near target**	90%	60%

Supplementary Table 1 | Summary of library preparation specifications for each cell line dilution.

Adapter Name	Sequences (5'-->3')
MA1	GATCTACACTCTTTCCCTACACGACGCTCTTCCGATCTAA*T
	/5Phos/TTAGATCGGAAGAGCACACGTCTGAACTCCAGTCAC
MA2	GATCTACACTCTTTCCCTACACGACGCTCTTCCGATCTAT*T
	/5Phos/ATAGATCGGAAGAGCACACGTCTGAACTCCAGTCAC
MA3	GATCTACACTCTTTCCCTACACGACGCTCTTCCGATCTAG*T
	/5Phos/CTAGATCGGAAGAGCACACGTCTGAACTCCAGTCAC
MA4	GATCTACACTCTTTCCCTACACGACGCTCTTCCGATCTAC*T
	/5Phos/GTAGATCGGAAGAGCACACGTCTGAACTCCAGTCAC
MA5	GATCTACACTCTTTCCCTACACGACGCTCTTCCGATCTTT*T
	/5Phos/AAAGATCGGAAGAGCACACGTCTGAACTCCAGTCAC
MA6	GATCTACACTCTTTCCCTACACGACGCTCTTCCGATCTTA*T
	/5Phos/TAAGATCGGAAGAGCACACGTCTGAACTCCAGTCAC
MA7	GATCTACACTCTTTCCCTACACGACGCTCTTCCGATCTTG*T
	/5Phos/CAAGATCGGAAGAGCACACGTCTGAACTCCAGTCAC
MA8	GATCTACACTCTTTCCCTACACGACGCTCTTCCGATCTTC*T
	/5Phos/GAAGATCGGAAGAGCACACGTCTGAACTCCAGTCAC
MA9	GATCTACACTCTTTCCCTACACGACGCTCTTCCGATCTGA*T
	/5Phos/TCAGATCGGAAGAGCACACGTCTGAACTCCAGTCAC
MA10	GATCTACACTCTTTCCCTACACGACGCTCTTCCGATCTGT*T
	/5Phos/ACAGATCGGAAGAGCACACGTCTGAACTCCAGTCAC
MA11	GATCTACACTCTTTCCCTACACGACGCTCTTCCGATCTGG*T
	/5Phos/CCAGATCGGAAGAGCACACGTCTGAACTCCAGTCAC
MA12	GATCTACACTCTTTCCCTACACGACGCTCTTCCGATCTGC*T
	/5Phos/GCAGATCGGAAGAGCACACGTCTGAACTCCAGTCAC
MA13	GATCTACACTCTTTCCCTACACGACGCTCTTCCGATCTCA*T
	/5Phos/TGAGATCGGAAGAGCACACGTCTGAACTCCAGTCAC
MA14	GATCTACACTCTTTCCCTACACGACGCTCTTCCGATCTCT*T
	/5Phos/AGAGATCGGAAGAGCACACGTCTGAACTCCAGTCAC
MA15	GATCTACACTCTTTCCCTACACGACGCTCTTCCGATCTCG*T
	/5Phos/CGAGATCGGAAGAGCACACGTCTGAACTCCAGTCAC
MA16	GATCTACACTCTTTCCCTACACGACGCTCTTCCGATCTCC*T
	/5Phos/GGAGATCGGAAGAGCACACGTCTGAACTCCAGTCAC

Supplementary Table 2 | UMI-containing adapters used for ligation during library preparation.

Primer Name	Sequences (5'-->3')
Universal Primer	AATGATACGGCGACCACCGAGATCTACACTCTTTCCCTACACGA
Index 1	CAAGCAGAAGACGGCATACGAGATTGTGACGTGTGACTGGAGTTCAGACGTG
Index 2	CAAGCAGAAGACGGCATACGAGATCCACTTAGGTGACTGGAGTTCAGACGTG
Index 3	CAAGCAGAAGACGGCATACGAGATCGGTTACAGTGACTGGAGTTCAGACGTG
Index 4	CAAGCAGAAGACGGCATACGAGATGATGGTCAGTGACTGGAGTTCAGACGTG
Index 5	CAAGCAGAAGACGGCATACGAGATGCCAAGTTGTGACTGGAGTTCAGACGTG
Index 6	CAAGCAGAAGACGGCATACGAGATAACCGAGAGTGACTGGAGTTCAGACGTG
Index 7	CAAGCAGAAGACGGCATACGAGATGTCCATACGTGACTGGAGTTCAGACGTG
Index 8	CAAGCAGAAGACGGCATACGAGATTGGCAGAAGTGACTGGAGTTCAGACGTG
Index 9	CAAGCAGAAGACGGCATACGAGATTTCGTTGGGTGACTGGAGTTCAGACGTG
Index 10	CAAGCAGAAGACGGCATACGAGATAAGGTGGTGTGACTGGAGTTCAGACGTG
Index 11	CAAGCAGAAGACGGCATACGAGATACAAGGACGTGACTGGAGTTCAGACGTG
Index 12	CAAGCAGAAGACGGCATACGAGATACGGAATGGTGACTGGAGTTCAGACGTG
Index 13	CAAGCAGAAGACGGCATACGAGATAACAACCGGTGACTGGAGTTCAGACGTG
Index 14	CAAGCAGAAGACGGCATACGAGATAACGCTTCGTGACTGGAGTTCAGACGTG
Index 15	CAAGCAGAAGACGGCATACGAGATACCTCACTGTGACTGGAGTTCAGACGTG
Index 16	CAAGCAGAAGACGGCATACGAGATACTGCCAAGTGACTGGAGTTCAGACGTG
Index 17	CAAGCAGAAGACGGCATACGAGATAGATTCGCGTGACTGGAGTTCAGACGTG
Index 18	CAAGCAGAAGACGGCATACGAGATAGTCTGTGGTGACTGGAGTTCAGACGTG
Index 19	CAAGCAGAAGACGGCATACGAGATATGTAGCCGTGACTGGAGTTCAGACGTG
Index 20	CAAGCAGAAGACGGCATACGAGATCAGAGCTAGTGACTGGAGTTCAGACGTG
Index 21	CAAGCAGAAGACGGCATACGAGATCATACAGGGTGACTGGAGTTCAGACGTG
Index 22	CAAGCAGAAGACGGCATACGAGATCCTTGATCGTGACTGGAGTTCAGACGTG
Index 23	CAAGCAGAAGACGGCATACGAGATCTAACTCCGTGACTGGAGTTCAGACGTG
Index 24	CAAGCAGAAGACGGCATACGAGATGGAACCATGTGACTGGAGTTCAGACGTG

Supplementary Table 3 | Indexed primers used for PCR during library preparation.
